# Supplementary material for: Fast and Sensitive Bacteria Detection by Boronic Acid Modified Fluorescent Dendrimer
Source: Sensors (Basel). 2021 Apr 30;21(9):3115. doi: 10.3390/s21093115 (PMC8124657; doi:10.3390/s21093115)
Supplement: Supplementary file 1 [file sensors-21-03115-s001.zip › sensors-1206528-supplementary.pdf]

sensors /

Supplementary

Fast and Sensitive Bacteria Detection by Boronic Acid Modified Fluorescent Dendrimer

Ayame Mikagi[1], Riho Tsurufusa[1], Yuji Tsuchido[1,2], Takeshi Hashimoto[1] and Takashi Hayashita[1,*]

[1] Department of Materials and Life Sciences, Faculty of Science and Technology, Sophia University, 7-1 Kioi-cho, Chiyoda-ku, Tokyo 102-8554, Japan; iris.14mls@eagle.sophia.ac.jp (A.M.); r-tsuruhusa-r43@eagle.sophia.ac.jp (R.T.); y-tsuchido@aoni.waseda.jp (Y.T.); t-hasimo@sophia.ac.jp (T.H.)

[2] Department of Life Science and Medical Bioscience, School of Advanced Science and Engineering, Waseda University (TWIns), 2-2 Wakamatsu-cho, Shinjuku-ku, Tokyo 162-8480, Japan

* Correspondence: ta-hayas@sophia.ac.jp; Tel.: +81-3-3238-3372

1. ^{1}H NMR Spectra

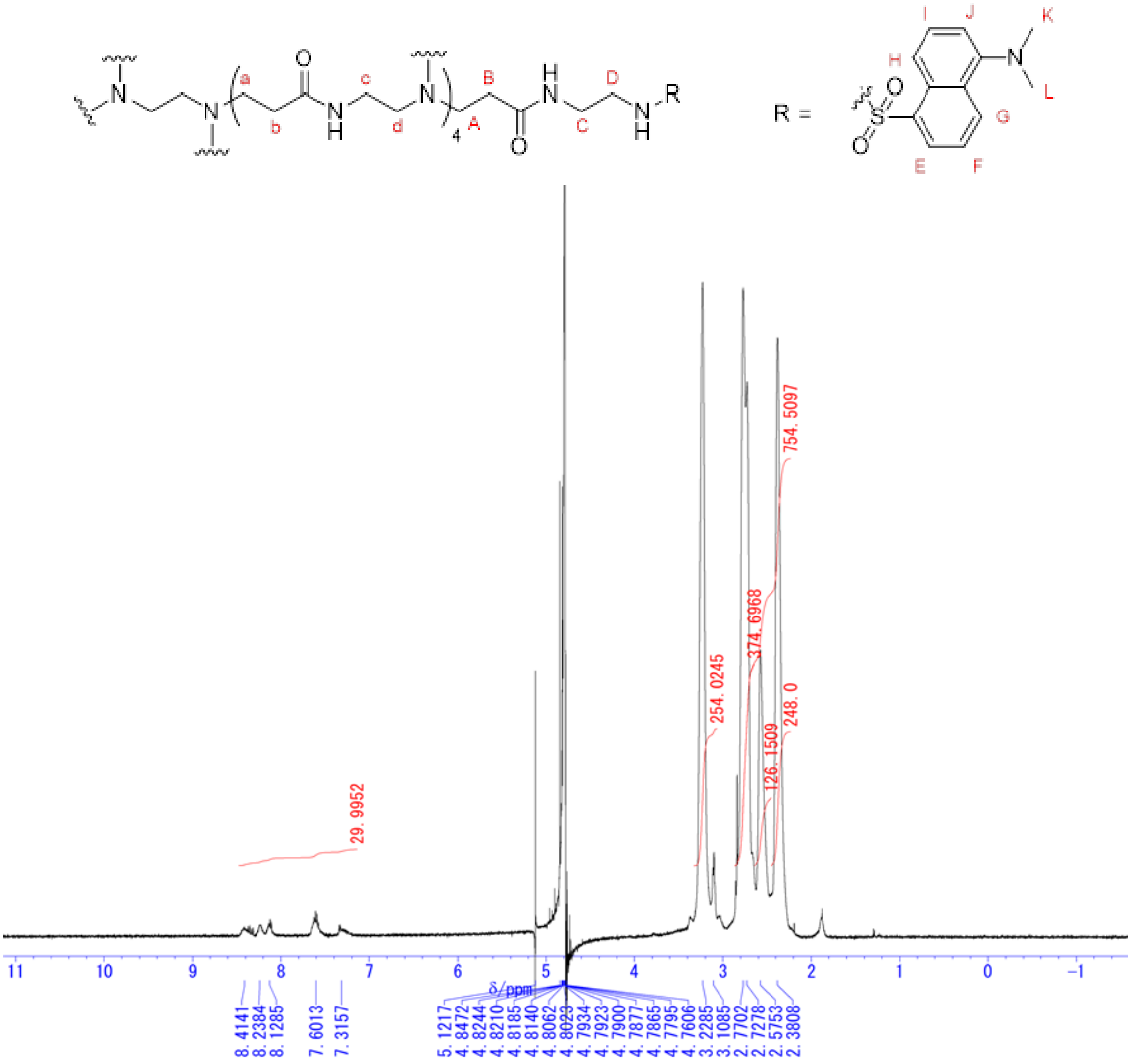

Figure S1. ^{1}H NMR spectrum of Dan-PAMAM (D_2O, the number of dansyl substitutions: 5). ^{1}H NMR (500 MHz, D_2O) δ(ppm): 8.41 (br, 30H, $H_{E\sim J}$), 8.24 (br, 30H, $H_{E\sim J}$), 8.13 (br, 30H, $H_{E\sim J}$), 7.60 (br, 30H, $H_{E\sim J}$), 7.32 (br, 30H, $H_{E\sim J}$), 3.23 (br, 248H, $H_{C,c}$), 2.77-2.72 (br, 496H, $H_{A,a,D,d}$), 2.57 (br, 496H, $H_{A,a,D,d}$), 2.38 (br, 248H, $H_{B,b}$); (E~J):(B+b) = 6M:248 (M: the number of dansyl substitutions): 29.9552:248 = 6M:248, M = 5.0.

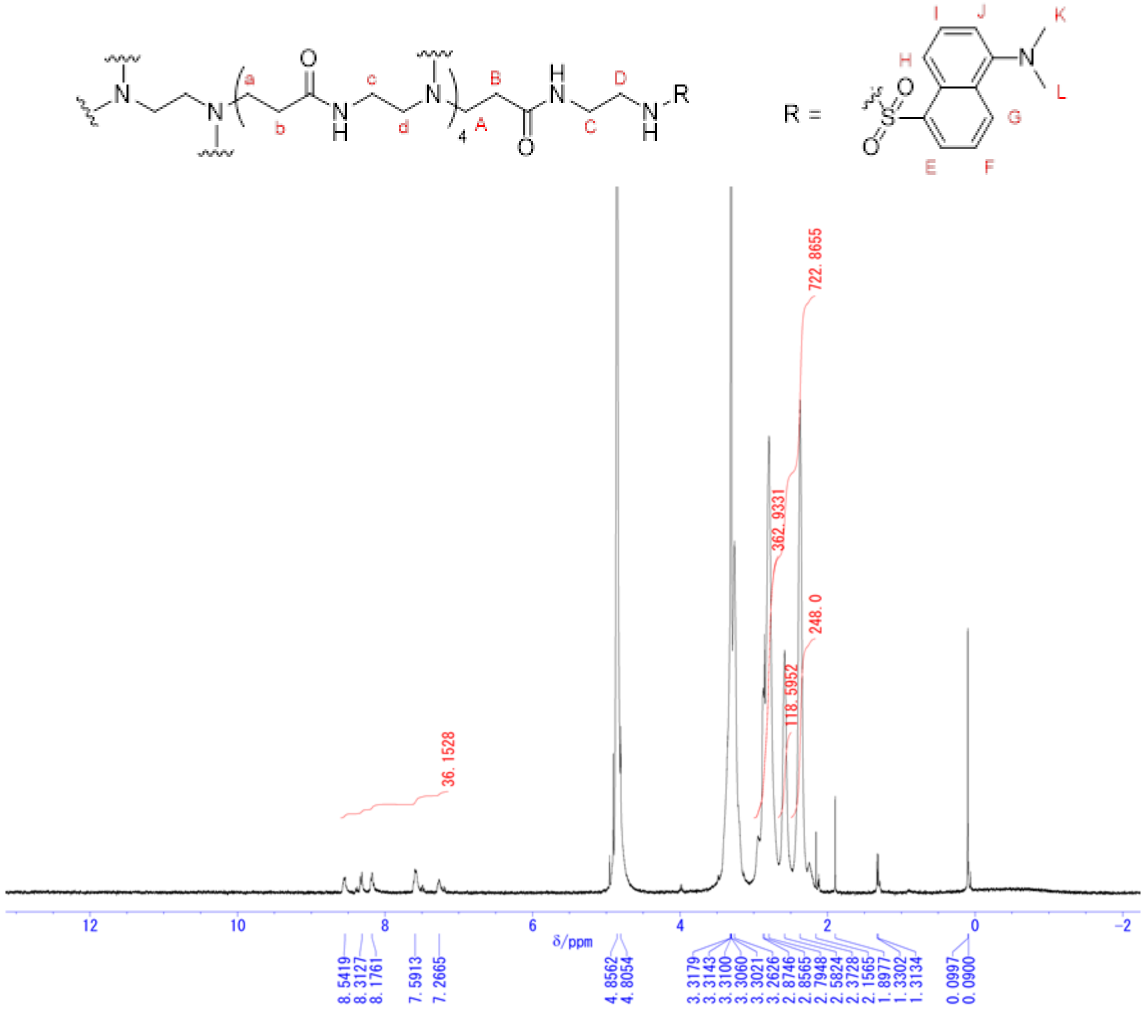

Figure S2. ^{1}H NMR spectrum of Dan-PAMAM (CD_3OD, the number of dansyl substitutions: 6). ^{1}H NMR (400 MHz, CD_3OD) δ(ppm): 8.54 (br, 36H, $H_{E\sim J}$), 8.31 (br, 36H, $H_{E\sim J}$), 8.18 (br, 36H, $H_{E\sim J}$), 7.59 (br, 36H, $H_{E\sim J}$), 7.27 (br, 36H, $H_{E\sim J}$), 3.26 (br, 248H, $H_{C,c}$), 2.87-2.80 (br, 496H, $H_{A,a,D,d}$), 2.58 (br, 496H, $H_{A,a,D,d}$), 2.37 (br, 248H, $H_{B,b}$)**;** (E~J):(B+b) = 6M:248 (M: the number of dansyl substitutions)**:** 36.1528:248 = 6M:248**,** M = 6.0**.**

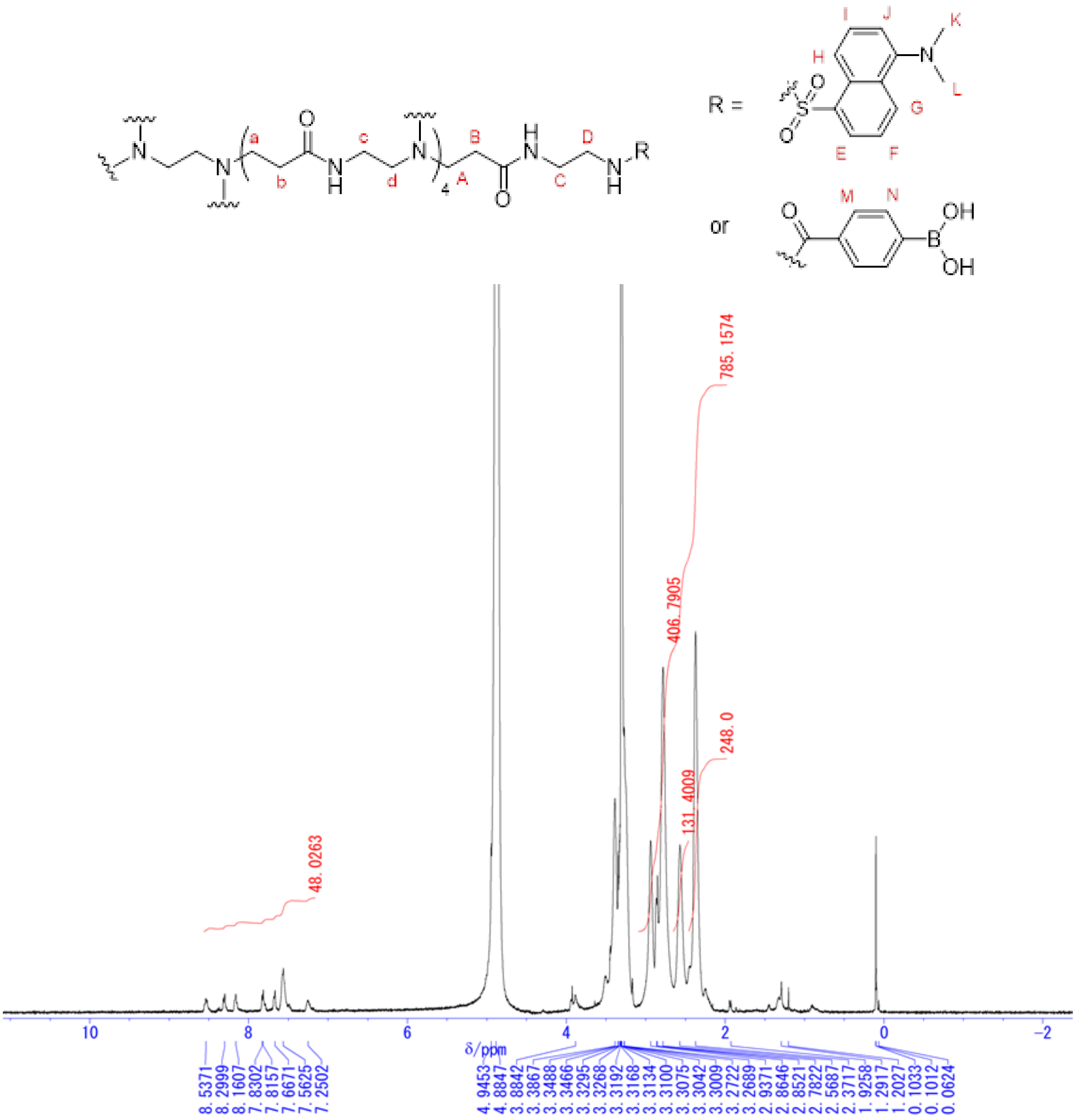

Figure S3. ^{1}H NMR spectrum of Dan-B3-PAMAM (CD_3OD). ^{1}H NMR (500 MHz, CD_3OD) δ(ppm): 8.54 (br, 42H, $H_{E\sim J,M,N}$), 8.30 (br, 42H, $H_{E\sim J,M,N}$), 8.16 (br, 42H, $H_{E\sim J,M,N}$), 7.83 (br, 42H, $H_{E\sim J,M,N}$), 7.67 (br, 42H, $H_{E\sim J,M,N}$), 7.56 (br, 42H, $H_{E\sim J,M,N}$), 7.25 (br, 42H, $H_{E\sim J,M,N}$), 3.35 (br, 248H, $H_{C,c}$), 2.93-2.78 (br, 496H, $H_{A,a,D,d}$), 2.57 (br, 496H, $H_{A,a,D,d}$), 2.37 (br, 248H, $H_{B,b}$)**;** (E~J,M,N):(B+b) = (4M+36):248 (M: the number of phenylboronic acid substitutions)**:** 48.0623:248 = (4M+36):248**,** M = 3.0**.**

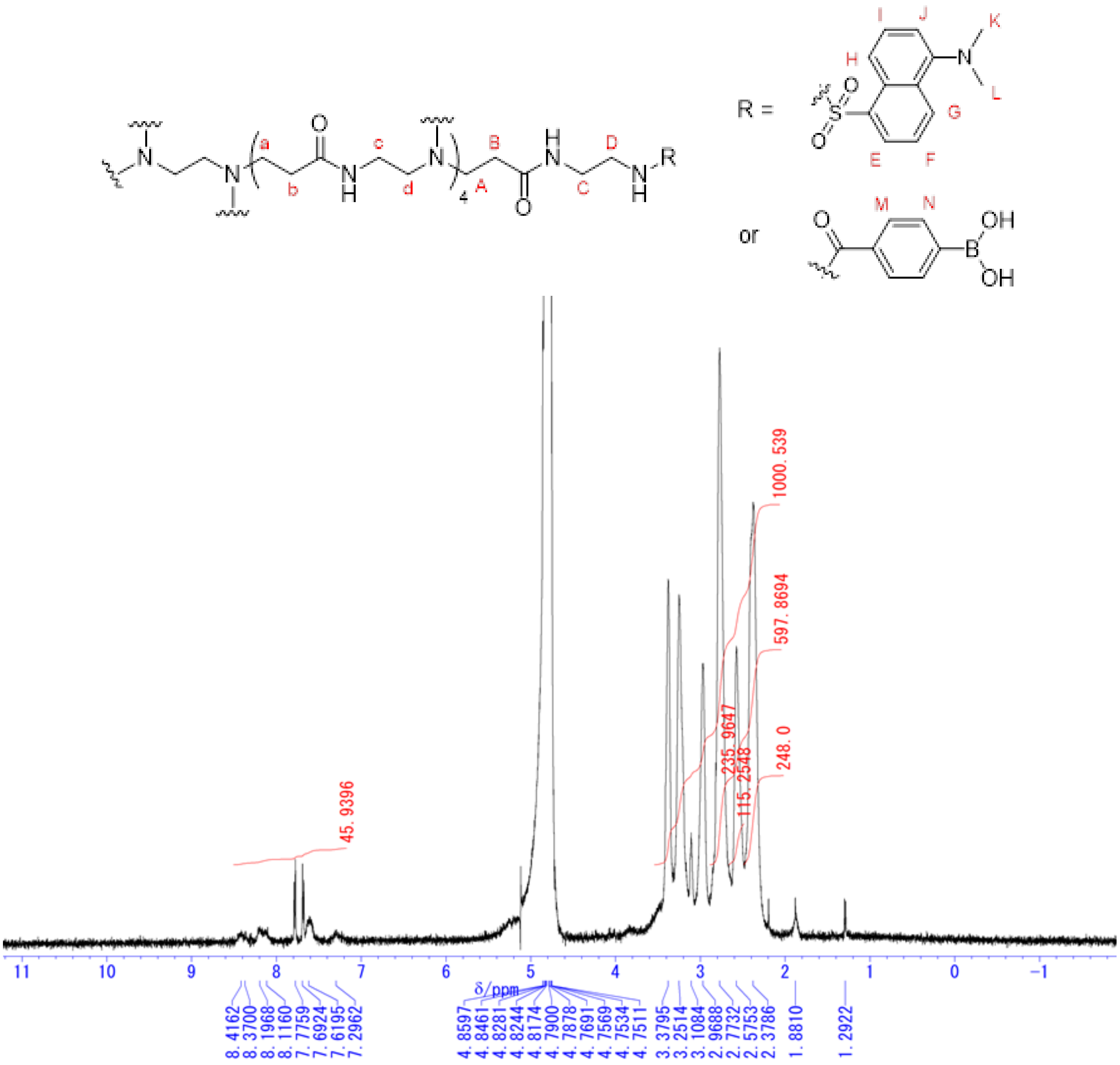

Figure S4. ^1H NMR spectrum of Dan-B4-PAMAM (D_2O). ^1H NMR (500 MHz, D_2O) δ(ppm): 8.42 (br, 46H, $H_{E\sim J,M,N}$), 8.37 (br, 46H, $H_{E\sim J,M,N}$), 8.20 (br, 46H, $H_{E\sim J,M,N}$), 8.12 (br, 46H, $H_{E\sim J,M,N}$), 7.78 (br, 46H, $H_{E\sim J,M,N}$), 7.69 (br, 46H, $H_{E\sim J,M,N}$), 7.62 (br, 46H, $H_{E\sim J,M,N}$), 7.30 (br, 46H, $H_{E\sim J,M,N}$), 3.38 (br, 744H, $H_{A,a,C,c,D,d}$), 3.25 (br, 744H, $H_{A,a,C,c,D,d}$), 3.11 (br, 744H, $H_{A,a,C,c,D,d}$), 2.93-2.78 (br, 744H, $H_{A,a,C,c,D,d}$), 2.57 (br, 744H, $H_{A,a,C,c,D,d}$), 2.37 (br, 248H, $H_{B,b}$); (E~J,M,N):(B+b) = (4M+30):248 (M: the number of phenylboronic acid substitutions): 45.9336:248 = (4M+30):248, M = 4.0.

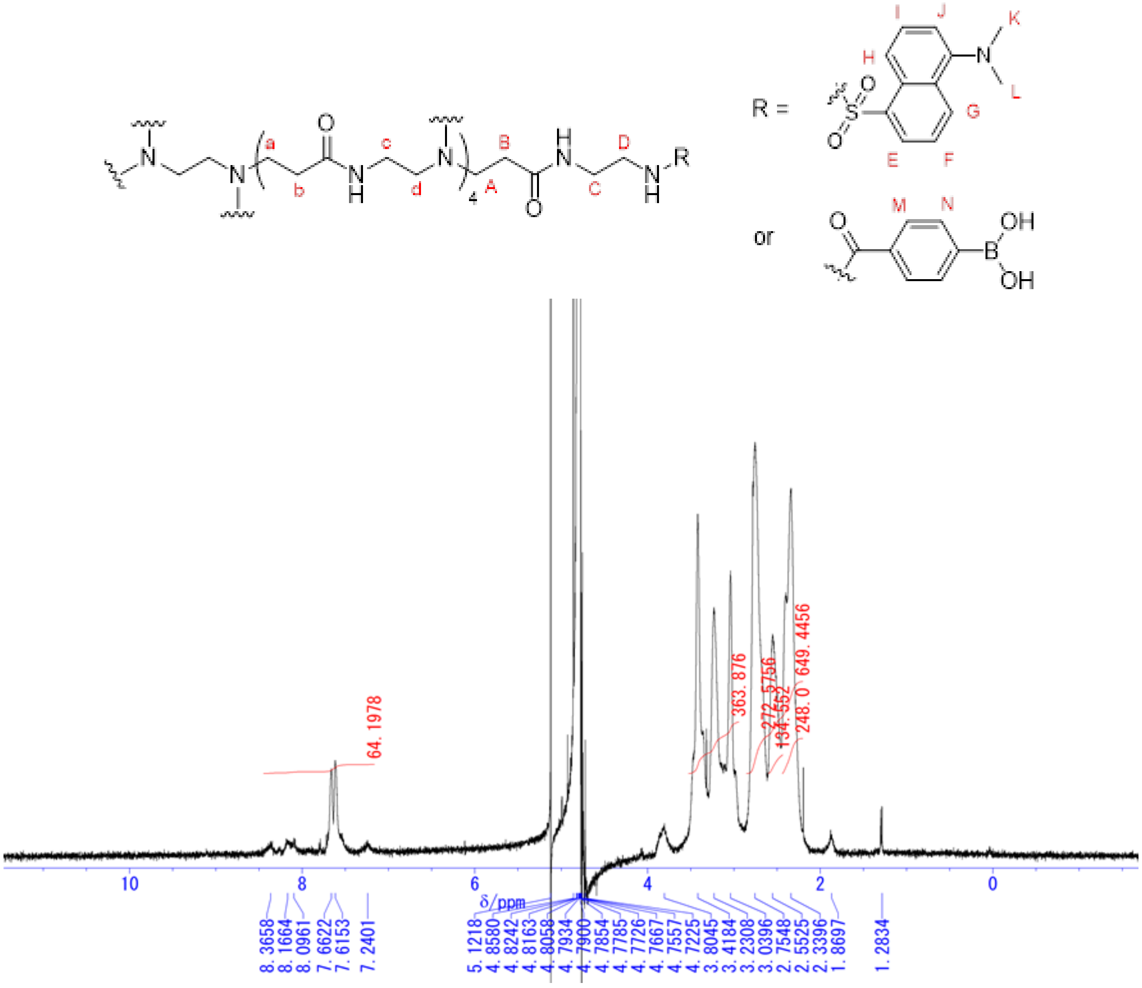

Figure S5. ^{1}H NMR spectrum of Dan-B8.5-PAMAM (D_2O). ^{1}H NMR (500 MHz, D_2O) δ(ppm): 8.37 (br, 46H, $H_{E\sim J,M,N}$), 8.17 (br, 46H, $H_{E\sim J,M,N}$), 8.10 (br, 46H, $H_{E\sim J,M,N}$), 7.66 (br, 46H, $H_{E\sim J,M,N}$), 7.62 (br, 46H, $H_{E\sim J,M,N}$), 7.24 (br, 46H, $H_{E\sim J,M,N}$), 3.42 (br, 744H, $H_{A,a,C,c,D,d}$), 3.23 (br, 744H, $H_{A,a,C,c,D,d}$), 3.04 (br, 744H, $H_{A,a,C,c,D,d}$), 2.75 (br, 744H, $H_{A,a,C,c,D,d}$), 2.55 (br, 744H, $H_{A,a,C,c,D,d}$), 2.34 (br, 248H, $H_{B,b}$); (E~J,M,N):(B+b) = (4M+30):248 (M: the number of phenylboronic acid substitutions): 64.1978:248 = (4M+30):248, M = 8.5.

2. Analytical Data

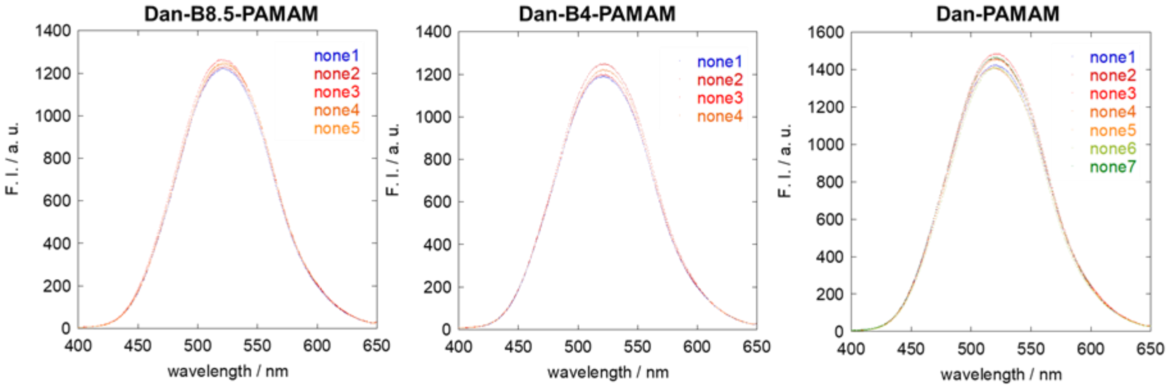

Figure S6. Fluorescence measurements of control samples in PBS buffer pH 7.4 (λ_{ex} = 330 nm, λ_{em} = 522 nm, [probe] = 3.3 μM).

Table S1. Fluorescence intensity of control samples at 522 nm in PBS buffer pH 7.4 (λ_{ex} = 330 nm, λ_{em} = 522 nm, [probe] = 3.3 μM). The entire spectra: see Figure S6.

	**Dan-PAMAM**	**Dan-B4-PAMAM**	**Dan-B8.5-PAMAM**
none1	1421	1188	1221
none2	1459	1249	1229
none3	1484	1197	1261
none4	1404	1219	1241
none5	1454		1248
none6	1408		
none7	1464		
F_{none} average	1442	1213	1240
SD	28.59	23.52	14.06

Table S2. Fluorescence intensity of Dan-B-PAMAMs in PBS buffer pH 7.4 (λ_{ex} = 330 nm, λ_{em} = 522 nm, [probe] = 3.3 μM, [bacteria] = 10^8 CFU·mL^{-1}).

S. aureus

	**Dan-PAMAM**	**Dan-B4-PAMAM**	**Dan-B8.5-PAMAM**
1	1356	1112	1058
2	1366	1133	1077
3	1360	1122	1065
Average	1361	1122	1067
SD	4.110	8.576	7.846

E. coli

	**Dan-PAMAM**	**Dan-B4-PAMAM**	**Dan-B8.5-PAMAM**
1	1315	1178	1088
2	1342	1120	1089
3	1349	1143	1113
Average	1335	1147	1097
SD	14.66	13.77	11.56

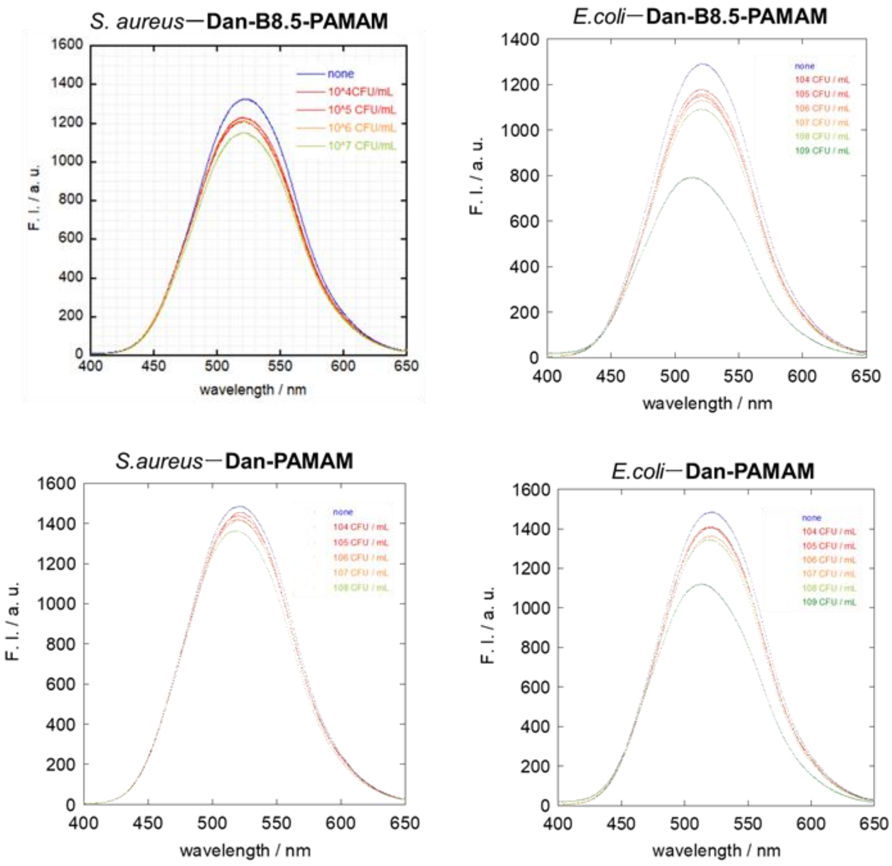

Figure S7. Examples of fluorescence spectra of the mixture between the probe and bacteria in PBS buffer pH 7.4 (λ_{ex} = 330 nm). [probe] = 3.3 μM, [*S. aureus*] = 10^4-10^8 CFU·mL^{-1} (Since Dan-B8.5-PAMAM showed visible aggregates in 10^8 CFU·mL^{-1}, it was not obtained.), [*E. coli*] = 10^4-10^9 CFU·mL^{-1}. Entire lists of fluorescent intensity: see Table S1-S3.

Table S3. Fluorescence intensity of the mixture between the probe and bacteria in PBS buffer pH 7.4 (λ_{ex} = 330 nm, λ_{em} = 522 nm). [probe] = 3.3 μM, [*S. aureus*] = 10^4-10^7 or 10^8 CFU·mL^{-1}, [*E. coli*] = 10^4-10^9 CFU·mL^{-1}. Results about F_{none}: see Table S1, 10^8 CFU·mL^{-1}: see Table S2. Welch's t-test was used to compare F_{none} and each concentration. Differences were calculated with two-side test with an alpha level of 0.05. Asterisk was considered as significant difference ($p < 0.05$).

Dan-B8.5-PAMAM with *S. aureus*

	F_{none}	10^4 CFU mL^{-1}	10^5 CFU mL^{-1}	10^6 CFU mL^{-1}	10^7 CFU mL^{-1}
1	1325	1209	1227	1212	1151
2	1299	1254	1239	1253	1139
3	1282	1229	1243	1245	1161
4	1279				
5	1284				
Average	1294	1231	1236	1237	1150
SD	17.06	18.41	6.799	17.75	8.994
p value	-	0.018*	0.001*	0.021*	0.00001*

Since the measurements of Dan-B8.5-PAMAM with *S. aureus* were conducted on another day, control (F_{none}) was newly obtained for the experiments.

Dan-B8.5-PAMAM with *E. coli*

	10^4 CFU mL^{-1}	10^5 CFU mL^{-1}	10^6 CFU mL^{-1}	10^7 CFU mL^{-1}	10^9 CFU mL^{-1}
1	1127	1131	1090	1130	777.1
2	1149	1177	1157	1128	819.6
3	1136	1157	1119	1149	764.3
Average	1137	1155	1122	1136	787.0
SD	9.031	18.83	27.43	9.463	23.64
p value	0.00006*	0.0010*	0.016*	0.00007*	0.0002*

p value of 10^8 CFU·mL^{-1}: 0.00006*.

Dan-PAMAM with *S. aureus*

	10^4 CFU mL^{-1}	10^5 CFU mL^{-1}	10^6 CFU mL^{-1}	10^7 CFU mL^{-1}
1	1436	1427	1420	1429
2	1419	1453	1407	1453
3	1313	1400	1418	1412
Average	1389	1427	1415	1431
SD	54.42	21.64	5.715	16.82
p value	0.302	0.466	0.064	0.546

Dan-PAMAM with *E. coli*

	10^4 CFU mL^{-1}	10^5 CFU mL^{-1}	10^6 CFU mL^{-1}	10^7 CFU mL^{-1}	10^9 CFU mL^{-1}
1	1465	1423	1408	1401	988.9
2	1397	1420	1399	1383	1065
3	1408	1410	1404	1362	1097
Average	1423	1418	1404	1382	1050
SD	29.80	5.558	3.682	15.94	45.34
p value	0.490	0.088	0.016*	0.010*	0.003*

p value of 10^8 CFU·mL^{-1}: 0.0003*.

Table S4. Absorbance changes by MTT assay (λ_{abs} = 560 nm), [probe] = 3.3 μM, [*S. aureus*] = 3.0 × 10^8 CFU·mL^{-1}, [*E. coli*] = 3.0 × 10^8 CFU·mL^{-1}. Welch's t-test was used to compare control and each probe. Differences were calculated with two-side test with an alpha level of 0.05. Asterisk was considered as significant difference ($p < 0.05$).

S. aureus

	**control (PBS)**	**PAMAM (COOH)**	**PAMAM (NH_2)**	**Dan-PAMAM**	**Dan-B4-PAMAM**	**Dan-B8.5-PAMAM**
1	2.6933	2.6966	2.6962	2.7133	2.7125	2.6991
2	2.6752	2.6796	2.6752	2.6814	2.6756	2.6764
3	2.6806	2.6786	2.6740	2.6822	2.6803	2.6770
Average	2.6830	2.6849	2.6818	2.6923	2.6895	2.6842
SD	7.5870×10^{-3}	8.2597×10^{-3}	0.010194	0.014853	0.016400	0.010562
p value	-	0.823	0.898	0.490	0.651	0.908

E. coli

	**control (PBS)**	**PAMAM (COOH)**	**PAMAM (NH_2)**	**Dan-PAMAM**	**Dan-B4-PAMAM**	**Dan-B8.5-PAMAM**
1	3.2752	3.1024	3.0644	3.2952	3.3053	1.7634
2	2.5414	2.5516	1.8659	2.4474	2.2686	0.8538
3	2.6580	2.5772	2.0177	2.4674	2.4305	1.2944
Average	2.8249	2.7437	2.3160	2.7367	2.6681	1.3039
SD	0.32198	0.25383	0.53281	0.39503	0.45537	0.37140
p value	-	0.794	0.325	0.819	0.713	0.012*

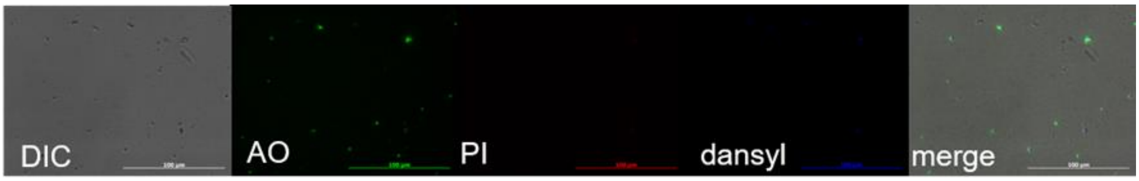

Figure S8. Fluorescence microscopy images of *S. aureus* with Dan-PAMAM. *S. aureus* (10^8 CFU·mL^{-1}) was stained with AO and PI, AO: λ_{ex} = 500 nm, PI: λ_{ex} = 530 nm, Dan-PAMAM: λ_{ex} = 330 nm. [Dan-PAMAM] = 3.3 μM. The images suggested that Dan-PAMAM did not form bonds with bacteria and any damaged bacteria were not observed.
